# Supplementary material for: Bioinspired nacre-like alumina with a bulk-metallic glass-forming alloy as a compliant phase
Source: Nat Commun. 2019 Feb 27;10:961. doi: 10.1038/s41467-019-08753-6 (PMC6393428; doi:10.1038/s41467-019-08753-6)
Supplement: Supplementary file 1 — Supplementary Information [file 41467_2019_8753_MOESM1_ESM.pdf]

Supplementary Information

Bioinspired Nacre-Like Alumina with a Bulk-Metallic Glass-Forming Alloy as a Compliant Phase

A. Wat, J.-I. Lee, C. W. Ryu, B. Gludovatz, J. Y. Kim, A. P. Tomsia, T. Ishikawa, J. Schmitz, A. Meyer, M. Alfreider, D. Kiener, E. S. Park and R. O. Ritchie

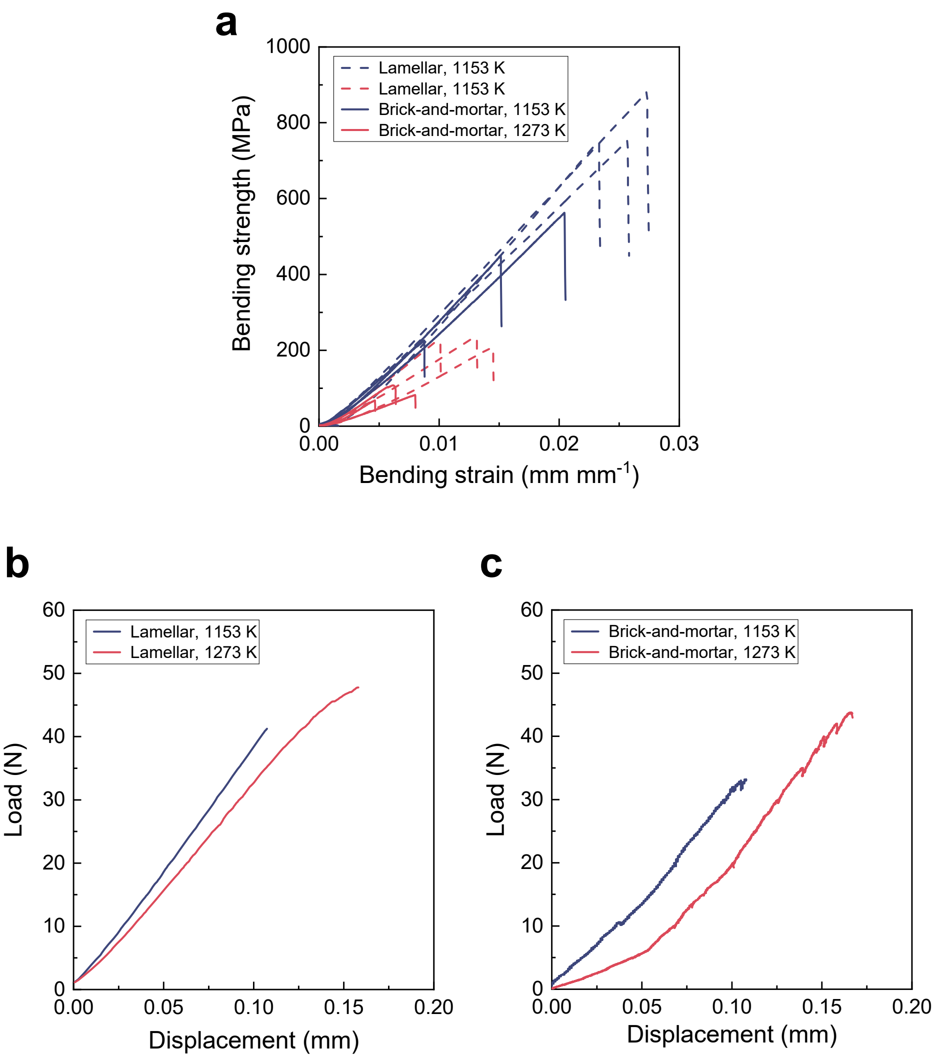

Supplementary Figure 1. Macroscale Mechanical Testing Data. (a) Stress-strain curves for three-point bending flexural strength tests indicate how microstructure and infiltration temperature affects the strength and ductility of the materials. (b, c) Representative load-displacement curves for fracture toughness tests conducted on lamellar and brick-and-mortar samples infiltrated at 1153 K and 1273 K.

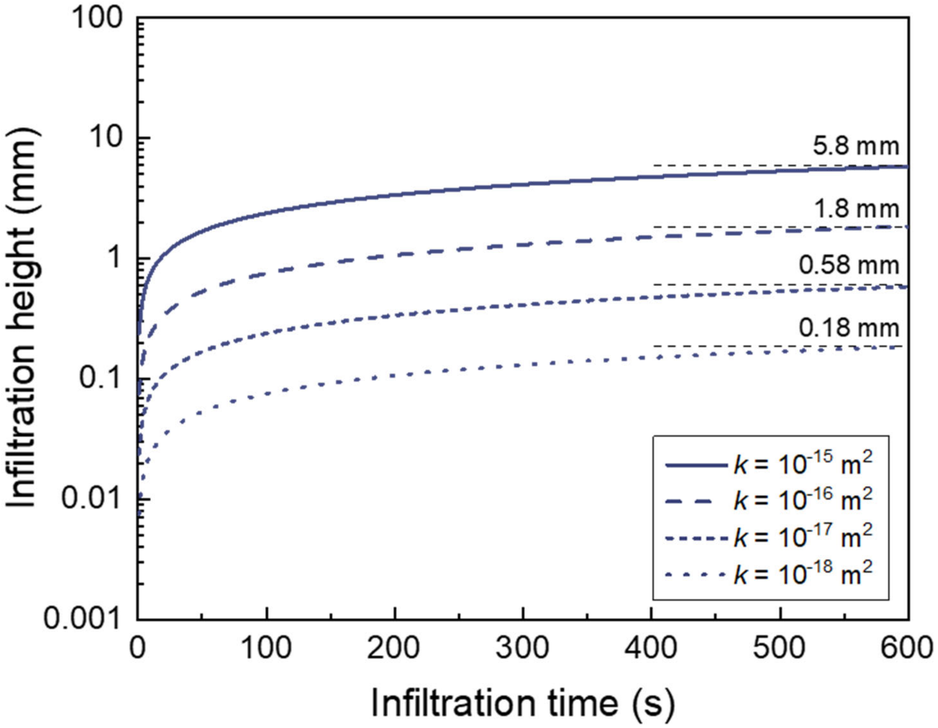

Supplementary Figure 2. Estimated infiltration heights of bulk-metallic glass melts into alumina. Estimated infiltration heights of the BMG melt at 1153 K, assuming different values of permeability of the brick-and-mortar scaffold in the range from 10^{-18} m^2 to 10^{-15} m^2. The calculation shows that the melt-infiltration of the nacre-like scaffolds in this study can be completed within 10 min despite the high viscosity of the BMG melt.

Supplementary Note 1. Work of adhesion calculations

The excellent wettability allowed spontaneous infiltration of the nacre-like alumina scaffolds at very rapid rates (10 min hold time) without the need for applied pressure. To characterize the critical characteristics to achieve this infiltration behavior, the thermophysical properties and wetting behavior of the BMG melt were evaluated. The surface tension of the Zr-based BMG at its melting point was estimated at 1.24 $N \cdot m^{-1}$, making it comparable to other Zr-based BMGs like Vitreloy 1 or Vitreloy 106.[1] Interestingly, this value showed only a small deviation (~6%) from the calculated value based on the rule of mixtures (1.32 $N \cdot m^{-1}$) of the surface tension of the pure components (1.48 $N \cdot m^{-1}$, 1.35 $N \cdot m^{-1}$, 0.91 $N \cdot m^{-1}$, 0.87 $N \cdot m^{-1}$ and 1.1 $N \cdot m^{-1}$, respectively for Zr, Cu, Ag, Al, and Be at their melting point).[2] These estimates imply that the BMG melt did not display significant surface segregation, as is often found in molten alloys comprised of elements with low surface tension.[1] Moreover, it indicates that the wettability of the system was not affected by any segregation or evaporation of elements with low vapor pressure like Ag. Based on this assumption, the strength of bonding between the BMG melt and the alumina can be derived from the surface tension, γ, evaluated from Figure 1a and the final contact angles, θ, measured by the sessile-drop method (Fig. 2). Specifically, the alumina/BMG melt bond strength can be estimated by calculating the work of adhesion (W_i), *i.e.*, the energy required to reversibly separate the two materials, *viz*:

$$W_i = \gamma(1 + \cos\theta) , \quad (1)$$

which yields values of W_i of 2.43 $J \cdot m^{-2}$ and 2.33 $J \cdot m^{-2}$ at 1153 K and 1273 K, respectively. These values are much higher than those for non-reactive metal/Al_2O_3 couples (0.2 – 1.5 $J \cdot m^{-2}$)[3], indicating the strong chemical interaction between the BMG melt and alumina. This confirms how reactive wetting of alumina by the BMG melt can induce spontaneous melt-infiltration into the nacre-like ceramic scaffolds without the need for external pressure.

Supplementary Note 2. Capillary pressure calculations

The spontaneous melt-infiltration is driven by capillary pressure (ΔP), where:

$$\Delta P = \frac{4\gamma\cos\theta}{D_e} , \quad (2)$$

and where D_e is the equivalent diameter of pores in the scaffold. The equivalent diameter, which is a function of reinforcement size and porosity, is equal to the capillary diameter for a single cylindrical tube. Considering that the pore structure in the nacre-like scaffolds is anisotropic, which is similar to a fibrous preform, we use here the relationship derived for fibrous preforms to describe the equivalent diameter of the nacre-like alumina scaffolds:[4]

$$D_e = \frac{4}{F}\frac{e}{(1-e)} D_{Al2O3} , \quad (3)$$

where F, e, and D_{Al2O3} are the form factor, porosity and average thickness of lamellar in the scaffolds, respectively. The form factor, which is dependent on the flow direction of a liquid in a scaffold, is 4 for the flow parallel and 2 for the flow perpendicular to fiber alignment.[4] Assuming a value of F = 3, which is the average between these two boundary cases, and considering the measured alumina thickness and volume fraction shown in Figure 4 of the main text, the equivalent diameters of the lamellar and brick-and-mortar scaffolds were estimated to be 17.5 µm and 7.5 µm, respectively. The estimate is reasonable in that those values are close to the thickness of the metallic mortar (13.3 ± 4.7 µm and 4.8 ± 4.2 µm, respectively) in the alumina/glass materials. The capillary pressures induced on the BMG melt were estimated as 277 kPa and 270 kPa for the lamellar scaffolds and 642 kPa and 619 kPa for the brick-and-mortar scaffolds at 1153 K and 1273 K, respectively. The pressure values at both temperatures were similar due to the weak temperature dependence of the surface tension and final contact angle (Figs. 1a,2). The result is that we achieve spontaneous melt-infiltration because the capillary pressures induced by the BMG melt is the same or higher than the external pressure necessary for complete infiltration in other experiments. The estimated pressure is on the same order of magnitude as the external pressure required to fabricate particulate BMG matrix composites[5] (550 kPa, 50 vol.% Mo, Nb, and Ta, 30 - 200 µm in diameter), and two orders of magnitude higher than the external pressure required for the fiber-reinforced BMG matrix composites[6] (6.9 kPa, 80 vol.% W, 250 µm in diameter). Thus, the spontaneous infiltration of the nacre-like scaffolds with the BMG melt can be deemed to result from the high capillary pressure.

Supplementary Note 3. Calculations of the kinetics of infiltration

The viscosity of the BMG at its melting point was estimated to be 2.12 × 10^{-1} Pa·s, which is two orders of magnitude higher than that of pure components at their melting point (~10^{-3} Pa·s).[26,27] This high viscosity is typical for the BMGs with high glass-forming ability due to the small amount of free volume in the liquid state.[1,7] However, the higher viscosity is unfavorable for the melt-infiltration in that it delays complete infiltration, which is dependent on the fluid velocity in a scaffold. The infiltration kinetics between the BMG melt and nacre-like alumina scaffolds in this study can be evaluated by Darcy's law[8] assuming one-dimensional infiltration of the melt through the scaffold:

$$h^2 = \frac{2k\Delta P}{e\eta} t \quad , \tag{4}$$

where h is infiltration height, t is infiltration time, and k is permeability of a scaffold. Supplementary Figure 2 shows the infiltration height of the BMG melt at 1153 K under different values of permeability of the brick-and-mortar scaffold in the range from 10^{-18} m^2 to 10^{-15} m^2. The calculation indicates that the brick-and mortar scaffold, which has an actual infiltration height of 1.25 mm that is half of the width of the scaffold (2.5 mm), can be infiltrated within 10 min if the permeability of the brick-and-mortar scaffold is higher than 5 x 10^{-17} m^2. The estimated permeability is reasonable in that the scaffolds, with ceramic fraction of up to 80 vol.%, showed their permeability in the range from 10^{-14} m^2 to 10^{-12} m^2.[8–11] Thus, the melt-infiltration of the nacre-like scaffolds in this study can be completed within 10 min, despite the high viscosity of the BMG melt.

However, it should be noted that the permeability of a scaffold is proportional to the square of the reinforcement size.[8] In this study, the micron-scale brick-and-mortar scaffolds exhibited a lamellae thickness of 22 μm, whereas the brick-and-mortar structure of natural nacre is some two orders of magnitude finer, with a mineral thickness of ~250 nm.[12] Accordingly, synthetic nacre-like scaffolds made to this fine-scale would reflect the true architectural scale of the natural material but would be far more difficult to infiltrate; the nano-scale features would induce a four orders of magnitude lower permeability than for micron-scale structures, which would result in very slow kinetics of melt-infiltration using this BMG (Supplementary Fig. 2). Thus, for the future synthesis of nano-scale nacre-like hybrid materials by melt-infiltration, the length-scale of the

scaffolds, together with the viscosity and wettability of a molten alloy, will need to be carefully considered to design feasible processing routes.

Assuming the slowest case that the half of width (1.25 mm) of the brick-and-mortar scaffold was fully infiltrated by the BMG melt in 10 min at 1153 K, the minimum infiltration rate in this study can be estimated as 7.5 $mm \cdot hr^{-1}$. The pressureless infiltration rate of Zr-based BMG into the brick-and-mortar alumina structure is higher than that of $Al_{94.5}Mg_{5.5}$ alloy into 62 vol.% particulate alumina scaffolds (3.3 $mm \cdot hr^{-1}$ at 1173 K)[13]. Compared to the Al-Mg alloy which requires incubation time to wet the alumina due to the removal of the native oxide layer on the molten alloy, the BMG melt perfectly wets the alumina within several tens of seconds. Thus, the rapid infiltration in the alumina-BMG system is advantageous to control the interfacial strength that is affected by the chemical reaction at elevated temperatures.

Supplementary References

1. Mukherjee, S., Johnson, W. L. & Rhim, W. K. Noncontact measurement of high-temperature surface tension and viscosity of bulk metallic glass-forming alloys using the drop oscillation technique. *Appl. Phys. Lett.* **86,** 014104 (2004).
2. Keene, B. J. Review of data for the surface tension of pure metals. *Int. Mater. Rev.* **38,** 157–192 (1993).
3. Saiz, E., Cannon, R. M. & Tomsia, A. P. High-temperature wetting and the work of adhesion in metal/oxide systems. *Annu. Rev. Mater. Res.* **38,** 197–226 (2008).
4. Ahn, K. J., Seferis, J. C. & Berg, J. C. Simultaneous measurements of permeability and capillary pressure of thermosetting matrices in woven fabric reinforcements. *Polym. Compos.* **12,** 146–152 (1991).
5. Dandliker, R. B., Conner, R. D. & Johnson, W. L. Melt infiltration casting of bulk metallic-glass matrix composites. *J. Mater. Res.* **13,** 2896–2901 (1998).
6. Choi-Yim, H., Schroers, J. & Johnson, W. L. Microstructures and mechanical properties of tungsten wire/particle reinforced $Zr_{57}Nb_5Al_{10}Cu_{15.4}Ni_{12.6}$ metallic glass matrix composites. *Appl. Phys. Lett.* **80,** 1906–1908 (2002).
7. Mukherjee, S., Schroers, J., Zhou, Z., Johnson, W. & Rhim, W.-K. Viscosity and specific volume of bulk metallic glass-forming alloys and their correlation with glass forming ability. *Acta Mater.* **52,** 3689–3695 (2004).
8. Garcia-Cordovilla, C., Louis, E. & Narciso, J. Pressure infiltration of packed ceramic particulates by liquid metals. *Acta Mater.* **47,** 4461–4479 (1999).

9. Molina, J., Saravanan. R.A., Arpón, R., García-Cordovilla, C., Louis, E. & Narciso, J. Pressure infiltration of liquid aluminium into packed SiC particulate with a bimodal size distribution. *Acta Mater.* **50,** 247–257 (2002).

10. Rodríguez-Guerrero, A., Narciso, J., Louis, E. & Rodríguez-Reinoso, F. Decreasing the infiltration threshold pressure of Al–12wt% Si into alumina particle compacts by Sn or Pb layers. *Compos. Sci. Technol.* **68,** 75–79 (2008).

11. Mattern, A. Huchler, B., Staudenecker, D., Oberacker, R., Nagel, A. & Hoffmann, M. J. Preparation of interpenetrating ceramic–metal composites. *J Eur. Ceram. Soc.* **24,** 3399–3408 (2004).

12. Mayer, G. Rigid biological systems as models for synthetic composites. *Science* **310,** 1144 (2005).

13. Srinivasa Rao, B. & Jayaram, V. Pressureless infiltration of Al–Mg based alloys into Al_2O_3 preforms: mechanisms and phenomenology. *Acta Mater.* **49,** 2373–2385 (2001).
